# ALDH1A Inhibition Suppresses Colitis and Alters α4β7 Integrin Expression on Activated T Cells in *Mdr1a*^−/−^ Mice

**DOI:** 10.3390/nu15183883

**Published:** 2023-09-06

**Authors:** Audrey Seamons, Olesya Staucean, Jessica M. Snyder, Thea Brabb, Charlie C. Hsu, Jisun Paik

**Affiliations:** Department of Comparative Medicine, University of Washington, Seattle, WA 98195, USA; auds@uw.edu (A.S.); olesya@uw.edu (O.S.); snyderjm@uw.edu (J.M.S.); thea@uw.edu (T.B.); chuckhsu@uw.edu (C.C.H.)

**Keywords:** retinoic acid, ALDH1A enzymes, immunity, inflammatory bowel disease, mouse model

## Abstract

There are limited pharmacological treatment options for inflammatory bowel disease (IBD), and some of these options are expensive and administered by injection or infusion. Thus, new cheaper and easier (oral) treatment options are needed. ALDH1A enzymes produce retinoic acid that can affect intestinal diseases such as IBD by regulating immune cells in the gut. We previously demonstrated that an orally deliverable ALDH1A inhibitor, WIN 18,466, can suppress colitis in an acute mouse model of IBD. Here, we tested the efficacy of ALDH1A inhibition in a chronic mouse model of IBD. *Mdr1a*^−/−^ mice were treated with a diet containing WIN 18,446 starting 1 week prior to inducing colitis by *H. bilis* inoculation. Treatment was continued until the study end point and colitis was monitored based on clinical symptoms and confirmed by histological analysis. Immune cell phenotypes in colon-draining lymph nodes (cMLN) were analyzed. WIN 18,446 treatment reduced clinical symptoms and improved histopathologic colitis scores. This was associated with decreased expression of the gut homing integrin, α4β7, on T cells in cMLN; increased expression of CD103, a protein associated with tissue-resident memory T cells; and changes in dendritic cells, plasmacytoid dendritic cells and B cells in inhibitor-treated mice. ALDH1A inhibition broadly influences immune cells during colitis and is a potential new target for IBD treatment. Future studies will be needed to determine the efficacy of ALDH1A inhibition on active colitis and to evaluate its relative efficacy in comparison to approved drugs.

## 1. Introduction

There is no cure for inflammatory bowel disease (IBD), and thus, life-long management of symptoms is needed [1]. Although first-line therapies for IBD patients often consist of non-specific anti-inflammatory drugs [2], more specific approaches have been developed that target inflammatory immune modulators such as monoclonal antibodies or small molecules that block cytokine signaling, integrins and integrin receptors, and lymphocyte migration [1]. However, no single treatment is effective in all cases of IBD, likely due to the diverse genetic and environmental factors that control disease progression and development. In addition, many of these treatment options are expensive and cumbersome to administer due to the need for injection and infusion. Therefore, new drugs that can be more cheaply produced and more easily administered to control gut inflammation are urgently needed.

Retinoic acid (RA) is a metabolite of vitamin A (retinol) and is synthesized by the aldehyde dehydrogenase 1A (ALDH1A) enzyme family [3,4]. RA is known to be involved in gut inflammation through the regulation of epithelial barrier function [5,6,7], lymphocyte gut homing ability [8,9], T cell differentiation [9,10] and IgA production [9]. RA has dual functions in immunity; it can enhance both inflammatory and regulatory immune responses depending on its concentration and the context of other immune modulatory molecules [9,10,11]. In agreement with the dual role of RA in gut inflammation, there are reports for both beneficial and harmful effects of RA in IBD [11,12,13,14,15,16,17]. Nonetheless, there is support for the strategy of reducing RA levels to suppress gut inflammation. For example, a decreased catabolism of RA was associated with increased risk of ulcerative colitis (UC) in humans [18], and RA was elevated in the mucosa of UC patients with active disease [19]. In acute graft vs. host disease, RA was increased in the intestine, and RA reduction by the overexpression of an RA-catabolizing enzyme, or deleting an RA-producing enzyme, reduced inflammation [20].

Until recently, it has not been possible to study the effects of reduced RA on inflammation because there were no specific inhibitors to reduce RA synthesis in vivo. Our group has been using a strong and specific ALDH1A inhibitor, WIN 18,446, to study the role of ALDH1A enzymes in spermatogenesis [21,22]. Our group has also used this inhibitor to study colitis, and showed that WIN 18,446 treatment reduced RA synthesis in vivo and suppressed colitis in an acute mouse model of IBD using *Smad3^−/−^* mice [23]. WIN 18,446 treatment was associated with the decreased expression of the gut homing α4ß7 integrin on activated CD4^+^ T cells [23], which is intriguing because some of the most effective biologics for the treatment of IBD are monoclonal antibodies targeting α4ß7 integrin [1].

In this report, we determined if inhibiting RA synthesis can be efficacious in reducing colitis arising from different etiologies by utilizing two additional models of IBD, *Il10^−/−^* and *Mdr1a^−/−^* mice. *Il10*^−/−^ mice lack a major regulatory cytokine important in dampening inflammatory immune responses. *Mdr1a^−/−^* mice develop spontaneous colitis primarily due to a gut barrier defect, and immune abnormalities in these mice are likely secondary to the primary gut barrier defect [24]. This model has been used in multiple studies testing drug efficacy for IBD [25,26]. Both strains of mice can develop colitis spontaneously, but the onset of the disease varies depending on environmental factors, such as the presence of *Helicobacter* or whether or not housing facilities are specific-pathogen-free [27]. In this study, colitis was triggered by infection with *Helicobacter bilis* to synchronize disease onset and development [28] in order to evaluate drug efficacy.

## 2. Materials and Methods

### 2.1. Studies to Test Effects of ALDH1A Inhibition on Colitis

Two separate experiments were performed. Male *Il10^−/−^* mice (BALB/cAnNTac-*Il10^em7Tac^*, model # 15660-M, 5–6 wo, *n* = 30) were obtained for the first experiment and female *Mdr1a^−/−^* mice (FVB.129P2-*Abcb1a^tm1Bor^*N, model # MDR1A-M, 7–8 wo, *n* = 30) were obtained for the second experiment from Taconic. We chose to use female *Mdr1a^−/−^* mice for our study as we observed frequent fighting among male *Mdr1a^−/−^* mice in our previous experiences. The fighting led to single-animal housing, which has potential to impact disease progression due to stress. All mice were acclimated in our *Helicobacter*-free, specific-pathogen-free facility and fed a purified irradiated diet, AIN93M (TestDiet, St. Louis, MO, USA). They were housed in individually ventilated cages with autoclaved corncob bedding (5 mice/cage) and autoclaved acidified water (pH 2.4–2.8). After about a one-week acclimation period, fifteen mice were switched to an AIN93M diet containing WIN 18,446 (2 mg/g diet) [22]. All mice were infected with *Helicobacter bilis* by oral gavage [29] one week after the WIN 18,446 diet initiation. Mice were weighed weekly and clinical scores for colitis symptoms were determined twice per week after diarrhea was observed in the cages. Clinical scores were determined by summing fecal scores (normal, 0; moist, sticky or soft, 1; diarrhea, 2; blood in feces, 3) and anal condition scores (none, 0; mild, 1; moderate, 2; severe, 3; rectal prolapse, 4). Mice were euthanized by CO_2_ asphyxiation followed by cardiocentesis for serum collection if they met predetermined criteria (>20% weight loss from the baseline, clinical score > 4, or body condition score ≤ 2), or a predetermined study end point (50% of mice in one group euthanized due to severe colitis). At necropsy, the colon and cecum were fixed in 10% neutral buffered formalin and processed to generate paraffin blocks (IDEXX). Hematoxylin and eosin (H&E)-stained colon and cecum sections (IDEXX) were evaluated by a board-certified veterinary pathologist blinded to the experimental groups. Colitis scoring was based on previously reported methods and included scoring four sites (cecum and proximal, mid and distal colon) for inflammation severity and extent each on a 0–4 scale and hyperplasia and dysplasia also on a 0–4 scale, for a maximum possible score of 64 [28].

### 2.2. Serum Cytokines and Helicobacter Status

Serum Cytokines were determined by Eve Technologies (Calgary, AB, Canada) using their Mouse Cytokine Th17 12-Plex Discovery Assay^®^ Array. For analysis purposes, in samples where analytes were below the limit of detection and could not be extrapolated using the mathematical formula of the standard curve, values were assigned as half of the lowest value within the standard curve or half of the lowest extrapolated experimental value in the assay in the case where there were lower extrapolated experimental values.

Helicobacter infection status was determined from the feces. Feces from each cage was collected at the initiation of diet treatment before *H. bilis* infection and 1–2 week following *H. bilis* inoculation to confirm that mice were free of *Helicobacter* at the start of the experiment and were colonized after the infection by PCR as previously reported [28]. All *Helicobacter*-infected mice shed the bacteria by 2 weeks after the infection.

### 2.3. Immune Cell Characterization in Mdr1a^−/−^ Mice

At the study end point, the most proximal colon-draining mesenteric lymph node (cMLN) was dissected and single cell suspensions were generated [29]. This lymph node was selected because we are interested in the colon-specific immune response. In *H. bilis*-infected *Mdr1a*^−/−^ mice, this lymph node is very prominent and easy to collect. The remaining mesenteric lymph nodes were used to generate control staining samples. Lamina propria lymphocytes could not be evaluated in this study because the tissues were used for histologic determination of colitis scores. Total cMLN live cell numbers were determined by counting cells using a hemocytometer after trypan-blue staining, and subsets were determined by staining cells with combinations of fluorescently labeled antibodies using previously published methods [23]. Live/Dead Blue (Thermofisher Scientific) was used for dead cell exclusion for all stains. Two different staining panels were evaluated. One panel evaluated T cell populations using antibodies specific for the following antigens: TCRβ, CD4, α4β7, CD103, CD195, FoxP3, and RORγt. The other panel contained antibodies against more general immune antigens: TCRβ, B220, CD103, CD11c, CD11b, MHC II, Ly6C and Ly6G. All antibodies were purchased from either BD Biosciences, eBiosciences or BioLegend. Singly stained cells, unstained cells and fluorescence minus one (F-1) controls (where all antibodies/fluorochromes in the combination are included except one) were generated for each antibody/fluorochrome used in the stain combinations, except in the case of the T cell stain. In the T cell stain, F-1 controls were not created for anti-CD4 or anti-TCR antibodies that showed well-defined separation from other cells based on our previous experience. Singly stained control cells and unstained cells were used to make instrument and compensation settings. F-1 controls were compared to the fully stained sample to set the gating for sample analysis. Where antibodies do not mark an abundant cell population (such as for RORγt or CD11c) or if expression of the antigen on the cells is not well separated from background (such as α4β7), a brightly staining single control antibody using the same fluorochrome was used in addition to the single-stained control for the antibody in question for instrument/compensation settings. Fluorescence staining was evaluated on an LSR II (BD Biosciences).

Flow cytometry data were analyzed using FlowJo v10.8.1 and associated Plugins. For the T cell panel, traditional manual gating was used to identify subsets of interest (Figure S1). The T cell subsets were determined based on the expression of CD4, RORγt and FoxP3, and in addition, were evaluated for the expression of α4β7 integrin, CD103, and CD195 (also known as CCR5). T cell subsets were evaluated by manual gating as follows: CD4^+^ T cells (CD4^+^ TCRβ^+^), CD4^−^ T cells (CD4^−^ TCRβ^+^), Th17 T cells (RORγt^+^FoxP3^−^ CD4^+^ T cells), iTregs (RORγt^+^FoxP3^+^ CD4^+^ T cells) and Tregs (RORγt^−^FoxP3^+^ CD4^+^ T cells). There were few RORγt^+^ and/or FoxP3^+^ cells among CD4^−^ T cells, and therefore, CD103 and α4β7 integrin were not evaluated in CD4^−^ T cell subsets defined by RORγt or FoxP3. For both staining combinations, we used dimensionality reduction techniques in combination with population discovery analysis to identify cell subsets that were altered with WIN 18,446 treatment. For these analyses, FlowAI v2.3 was run on each sample using all uncompensated fluorescent parameters to remove low-quality events prior to downstream analyses. Dead cells and cell doublets were excluded based on Live/Dead Blue staining (positive cells are dead) and SSC-W vs. SSC-H parameters, respectively. A leukocyte gate was created based on FSC-A and SSC-A parameters. Cells were subsampled from gates indicated in figures using the DOWNSAMPLE plugin (v3.3). Cells from the individual samples were then concatenated (*n* = 8 samples/treatment, total 16 samples) after adding keywords for no treatment and WIN 18,446 treatment so that individual samples from each treatment group could be identified in downstream analyses. The dimensionality reduction technique, t-distributed stochastic neighbor embedding (t-SNE), was performed to identify global changes in the various immune cell populations by creating a two-dimensional visualization of cell populations in the concatenated sample. The analysis was performed on compensated fluorescent parameters excluding the channel used to identify Live/Dead cells as dead cells were removed. In the case where cell subsets were selected based on gating for TCR, the fluorescent parameter for TCR was also excluded. Options were selected in FlowJo as follows: learning configuration = Opt-SNE, KNN algorithm = Approximate (random projection forest—ANNOY), and the gradient algorithm = FFT Interpolation (Flt-SNE). Default settings were used for iterations (1000), perplexity (30) and learning rate (51,520). Population discovery analyses were performed using the Phenograph [30] Plugin (v2.4) associated with FlowJo on the same concatenated samples and the same fluorescent parameters as used in the t-SNE analysis with default Phenograph v2.4 settings (K = 30). Keywords/sample names were used to examine the percent contribution of each individual sample to the Phenograph-defined population in the concatenated sample (shown in figures as a percentage of the concatenated sample). These percentages were plotted by treatment group. Populations identified by Phenograph with less than 100 cells were excluded from further analysis. Another population that was low in abundance (less than 300 cells) and appeared to be mostly small, dying cells was also excluded from analysis.

### 2.4. Statistical Analyses

Student’s *T*-test was used to compare colitis severity between two groups. Log rank-sum was used for survival analysis. For pairwise comparisons, data with normal distributions were evaluated with Student’s T-test with Welch’s correction. If data were not normally distributed, transformation was attempted to normalize data. If data could not be transformed to a normal distribution, non-parametric (Mann–Whitney) tests were performed. Pairwise comparisons of no treatment vs. WIN 18,446 treatment percent contribution to concatenated samples of Phenograph populations were performed using Multiple T-test analysis followed by the False Discovery Rate (FDR) method to account for multiple comparisons. FDR was performed at the 5% FDR rate, and FDR-adjusted *p*-values are presented for comparisons considered a discovery. All statistical analyses were performed using GraphPad Prism 9.4.1 for Windows.

## 3. Results

### 3.1. ALDH1A Inhibition Significantly Reduces the Severity of Colitis in Mdr1a^−/−^ Mice but Not in Il10^−/−^ Mice

**Figure 1:**
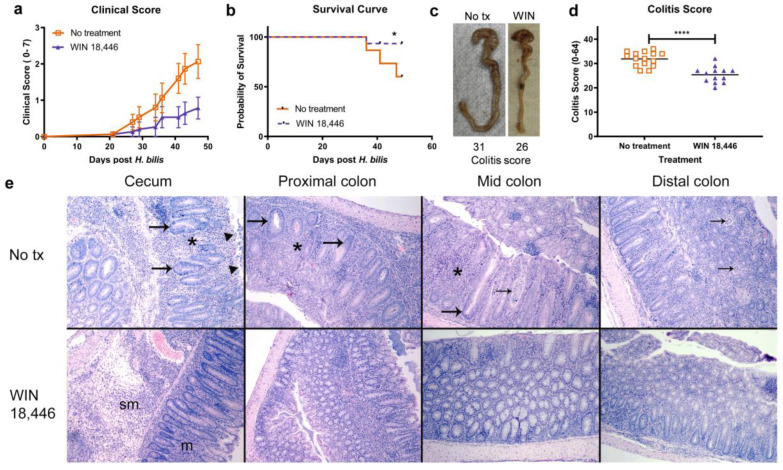
Suppression of colitis progression and severity by an ALDH1A inhibitor, WIN 18,446, in *Mdr1a^−/−^* mice. *Mdr1a^−/−^* mice were treated with and without WIN 18,446 (2 mg/g diet) one week prior to *H. bilis* inoculation; treatment (tx) was continued over the entire experiment. Clinical scores were determined twice a week once diarrhea was noted in a cage (**a**). Survival curve generated based on euthanasia of mice per predetermined criteria. The study was ended when 50% of one treatment group was euthanized due to severe colitis (**b**). Representative gross images of colons and ceca of mice in each treatment group. Images are shown for mice with median colitis scores per treatment group (**c**). Colitis severity scores as determined by histological analysis (**d**). Representative histological images of mouse ceca and colons shown in **c** (**e**). Mice from both groups have mucosal and submucosal inflammation and mucosal hyperplasia in the cecum, although untreated mice have more severe inflammation. The severe inflammation in mice in the no treatment group expands the lamina propria and separates the glands (*), and there is more severe epithelial cell hyperplasia (large arrow) and attenuation and erosion of the mucosal surface (triangles). In the colon, inflammation (*), hyperplasia (large arrow), and crypt inflammation (small arrows) and loss are also more severe in the no treatment group. m: mucosa, sm: submucosa. * *p* < 0.05, **** *p* < 0.0001.

To determine if ALDH1A inhibition reduces the severity of colitis, we treated *Mdr1a^−/−^* mice with WIN 18,446 in their diet for 1 week prior to infecting mice with the colitis trigger, *H. bilis*. Control mice (no treatment) received the same diet without WIN 18,446 for 1 week prior to the *H. bilis* trigger. Two weeks following the *H. bilis* infection, soft/sticky feces (diarrhea) was observed in five of the six cages. Mice with no treatment exhibited worse clinical symptoms, including diarrhea and erythema and swelling of the anus, compared to WIN 18,446-treated mice (Figure 1a). Fewer mice met early euthanasia criteria in the WIN 18,446 treatment group (1/15 with WIN 18,446 vs. 6/15 no treatment). The study was terminated at ~7 weeks following *H. bilis* infection as ~50% mice in the no treatment group were removed from the study due to severe disease (Figure 1b). At necropsy, WIN 18,446 treatment reduced thickening of the cecum and colon compared to no treatment (Figure 1c). Also, fewer of the mice in the treatment group had thickened colons and ceca (6 mice with WIN 18,446 treatment vs. 13 mice with no treatment). WIN 18,446 treatment significantly reduced histologically determined colitis severity scores (Figure 1d). A representative histology figure (Figure 1e) shows that WIN 18,446 treatment resulted in less severe mucosal and submucosal inflammation in the cecum and colon. In the untreated mice, more severe predominantly lymphocytic, plasmacytic and histiocytic inflammation expanded the lamina propria and submucosa in the cecum. In the colon, inflammation and hyperplasia were also less severe in the WIN 18,446 treatment group compared to no treatment. 

To determine if ALDH1A inhibition suppresses colitis in another mouse model of IBD with a different mechanism of disease development, we tested the efficacy of WIN 18,446 in *Il10^−/−^* mice using the same study design as *Mdr1a^−/−^* mice. Five days after *H. bilis* infection, we observed diarrhea in all cages. Approximately 50% of the mice (12/25) also showed erythema and swelling of the anus at this time point. The majority of infected mice deteriorated quickly showing severe weight loss (Figure S2a) with clinical scores of 2–3 by day 12 after *H. bilis* infection and were euthanized. Histological analysis of colon and cecum revealed that all mice had severe colitis regardless of treatment (Figure S2b). As we did not observe any protection from disease with WIN 18,446 treatment in the *Il10*^−/−^ mice, no further analyses were carried out in this model.

### 3.2. Changes in Serum IL17E and IL22 Are Associated with WIN 18,446 Treatment and Colitis in Mdr1a^−/−^ Mice

**Figure 2:**
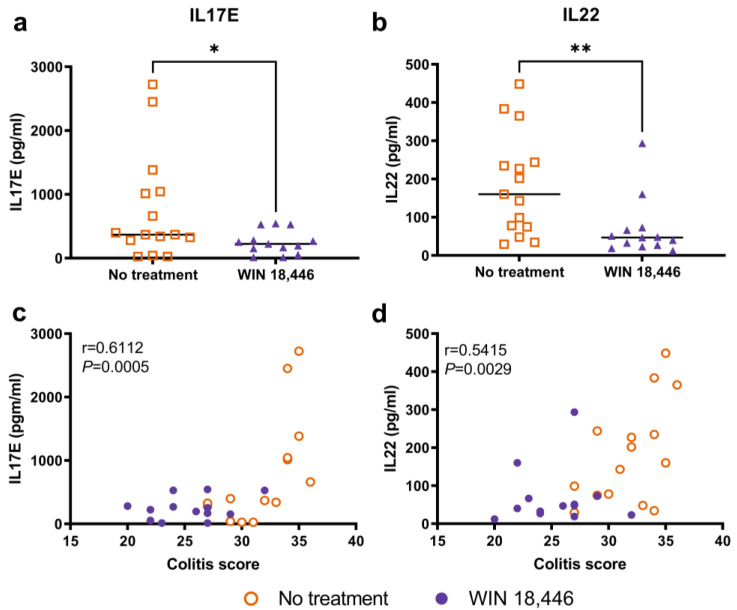
Serum cytokines altered with WIN 18,446 treatment and their correlation with colitis scores. Serum samples collected at study endpoint were evaluated with a panel of 12 Th17-related cytokines. IL17E (**a**) and IL22 (**b**) were significantly reduced in mice treated with WIN 18,446. Median value (horizontal bar) and significance (unadjusted Mann–Whitney) are indicated. * *p* < 0.05, ** *p* < 0.01. IL17E (**c**) and IL22 (**d**) were significantly correlated with colitis scores. Unadjusted Spearman r and associated *p*-values are indicated.

We had previously observed altered ratios of Th17 cells versus regulatory T cells with WIN 18,446 treatment in *Smad3^−/−^* mice, an acute IBD model [23]. The IL17 pathway is reported to play a significant role in colitis development in *Mdr1a^−/−^* mice [26]. Therefore, we determined if IL17 and related cytokines were globally altered with WIN 18,446 treatment during colitis in *Mdr1a^−/−^* mice. Of the 12 serum cytokines analyzed, only IL17E and IL22 were significantly different (unadjusted Mann–Whitney *p* = 0.0334 and 0.0044, respectively) between no treatment and WIN 18,446 treatment groups (Figure 2a,b). Serum values of these cytokines were moderately correlated (Spearman’s r = 0.6112 and 0.5415, respectively) with colitis scores (Figure 2c,d).

### 3.3. Colon-Draining MLN T Cell Subsets Are Altered with WIN 18,446 Treatment in Mdr1a^−/−^ Mice

We previously observed that RA synthesis inhibition via WIN 18,446 treatment decreased the expression of α4β7 in activated CD4^+^ T cells; this was associated with decreased colitis and increased survival in *Smad3^−/−^* mice [23]. To determine if RA synthesis inhibition induced similar changes in immune cells of *Mdr1a^−/−^* mice, we examined T cell populations in cMLN of *Mdr1a^−/−^* mice that made it to endpoint from the colitis experiment shown in Figure 1. Samples were evaluated by flow cytometry with a panel of T cell markers focused on Th17 and regulatory T cell (Treg) subsets, Th17, iTregs and Tregs, that we had previously studied in the *Smad3^−/−^* mouse model.

**Figure 3:**
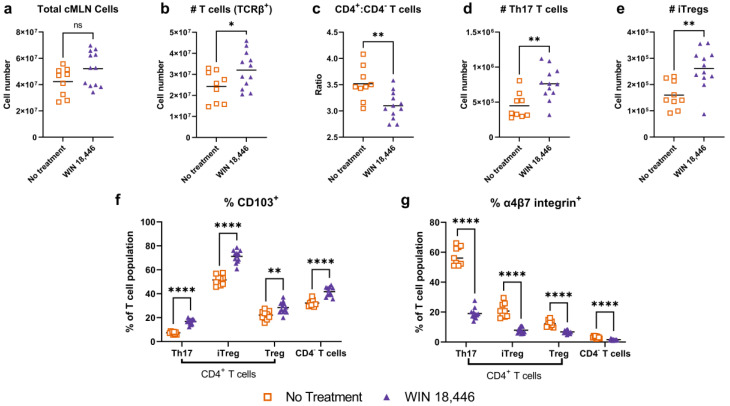
Changes in colon-draining MLN (cMLN) T cell subsets associated with WIN 18,446 treatment in the *Mdr1a*^−/−^ mouse model of IBD. Immune cells from *Mdr1a*^−/−^ mice cMLN were isolated at the study endpoint in the experiment shown in Figure 1. Total cMLN cell numbers as determined by manual counting (hemocytometer/trypan blue, (**a**)); T cell numbers (TCRβ^+^, (**b**)); ratios of CD4^+^ to CD4^−^ T cells (**c**); number of Th17 cells (CD4^+^ RORγt^+^FoxP3^−^ T cells, (**d**)); iTregs (CD4^+^ RORγt^+^FoxP3^+^ T cells, (**e**)); percent CD103^+^ of indicated T cell subsets (**f**); and percent α4ß7 integrin^+^ on T cell subsets (**g**) are shown. Bars on plots show mean values, and significance of pairwise comparisons (unadjusted unpaired t test with Welch’s correction) is indicated. #, number, ns = not significant, * *p* < 0.05, ** *p* < 0.01, **** *p* < 0.0001.

In the cMLN of *Mdr1a^−/−^* mice, there was an average increase in total immune cell numbers in mice treated with WIN 18,446 compared to no treatment, but this increase was not statistically significant (Figure 3a). Numbers of T cells were significantly increased with WIN 18,446 treatment (Figure 3b) though percentages of T cells were not significantly different between the treatment groups (Figure S3). Interestingly, there was a significantly decreased CD4^+^ to CD4^−^ T cell ratio in mice treated with WIN 18,446 (Figure 3c) due to increased cell numbers of CD4^−^ T cells in the WIN 18,446 treatment group and similar CD4^+^ T cell numbers between treatment groups (Figure S3). We also observed significantly increased numbers of CD4^+^ Th17 (Figure 3d) and iTreg (Figure 3e) cells in the WIN 18,446 treatment group, though percentages of these subsets were not significantly increased (Figure S3). We did not observe alterations in Th17 to iTreg ratios with WIN 18,446 treatment (Figure S3).

We noted changes in CD103 and α4β7 integrin in all T cell subsets examined. The percent of T cell subsets expressing CD103 was significantly increased (Figure 3f) with the magnitude of change being highest for Th17 cells (a 2.29-fold increase vs. 1.38-, 1.28- and 1.30-fold increases in iTreg, Tregs and CD4^−^ T cells, respectively). In contrast, the percent of cells expressing α4β7 integrin was significantly decreased (Figure 3g). The magnitude of this decrease was also largest among Th17 T cells with a 3.03-fold decrease (vs. 2.66-, 1.83- and 2.05-fold decreases in iTreg, Tregs and CD4^−^ T cells, respectively).

In addition to manual gating, we used population discovery methods to identify changes in T cell populations associated with WIN 18,446 treatment. We used Phenograph to distinguish populations based on similarities of protein expression, as identified by staining with the antibody panel, in a concatenated sample containing the same number of T cells from each individual sample of both treatment groups (Figure S4a). Phenograph identified 31 populations. Of the 31 populations identified by Phenograph, 24 were significantly altered with WIN 18,446 treatment. However, 15 of these 24 populations were CD4^+^ T cells that did not express any of the other markers in the staining panel (negative for RORγt, FoxP3, CD195, CD103, and α4β7) and had been differentiated by Phenograph based on varied fluorescence levels of the markers in the “negative” range where spreading error was large. Of the 24 populations, 2 were CD4^−^ T cells that did not express any other markers. Six of the populations could be separated based on markers other than CD4 expression (see Figure S5a,b). Similar to the manual gating analysis, populations expressing CD103 (Pheno 4 and 24) were increased with WIN 18,446 treatment while T cells expressing α4β7 integrin were decreased (Pheno 20 and 28). CD4^+^FoxP3^+^CD103^−^ (Pheno 9) cells were also decreased with WIN 18,446 treatment. Decreases were also observed in a CD4^−^ T cell population expressing mid-levels to no CD103. These changes were also visualized using another dimensionality reduction technique, tSNE (t-distributed stochastic neighbor embedding) analysis (Figure S5c).

### 3.4. Colon-Draining MLN Leukocyte Cell Subsets Are Altered with Treatment with WIN 18,446 in Mdr1a^−/−^ Mice

We evaluated changes in more general populations of leukocytes by performing flow cytometry on cMLN of *Mdr1a^−/−^* mice with a second panel of antibodies: TCRβ, B220, CD103, CD11c, CD11b, MHC II, Ly-6C and Ly6G. Because we were not certain a priori which of the leukocyte subsets might be altered in response to WIN 18,446 treatment, we used population discovery methods (Phenograph and tSNE) to evaluate changes in immune cells expressing these markers with WIN 18,446 treatment. Phenograph analysis was performed on two different concatenated samples composed of equal numbers of either (1) leukocytes or (2) TCRβ-negative (TCRβ^−^) cells (see Figure S4b for gating and subsampling strategies).

**Figure 4:**
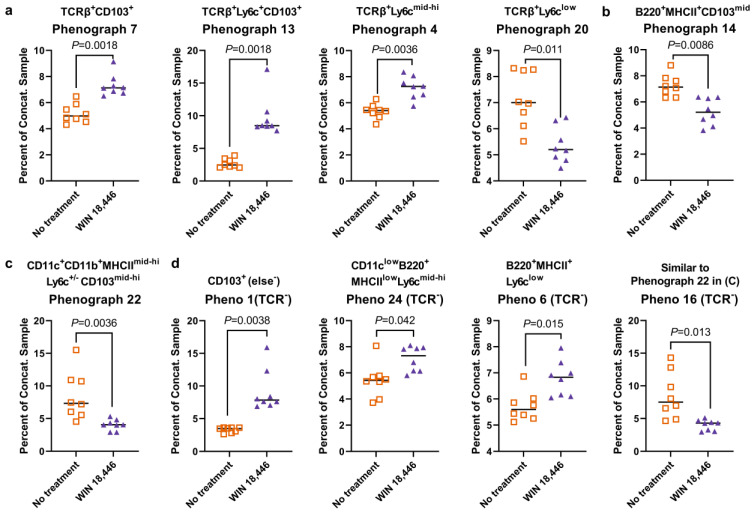
Changes in general immune cell subsets in colon-draining MLN (cMLN) with WIN 18,446 treatment in the *Mdr1a*^−/−^ mouse model of IBD. Leukocytes from *Mdr1a*^−/−^ mouse cMLN were isolated at the study endpoint in the experiment shown in Figure 1. Two concatenated samples (*n* = 8 samples/treatment, total 16 samples) composed of leukocytes or TCRβ^−^ leukocytes were evaluated using Phenograph. The percent contribution of each sample to the concatenated sample between the no treatment and WIN 18,446-treated groups were compared using multiple T-tests/FDR analysis. Among the concatenated leukocyte sample, four T cell populations (**a**), one B cell (**b**), and one DC (**c**) population were significantly altered with WIN 18,446 treatment. Defining phenotypes based on marker expression of each population are indicated in the text above each graph. Among the concatenated TCRβ^−^ leukocytes, four populations were identified to be significantly altered by WIN 18,446 treatment (**d**). FDR-adjusted (5%) *p*-values are shown for each comparison (**a**–**d**).

From the concatenated leukocyte sample, Phenograph identified 28 populations. Of the 28 populations, 6 were identified as discoveries (FDR adjusted *p*-values, *p* < 0.05). Four of these were T cell populations with variable staining for Ly6C and CD103 (Figure 4a), three of which were increased with WIN 18,446 treatment while a TCRβ^+^Ly6clow population was decreased. Additionally, WIN 18,446 treatment decreased a CD103^low^ B cell population (Figure 4b) and a CD11b^+^ dendritic cell (DC) population (Figure 4c and Figure S6).

We identified other non-T cell leukocyte populations altered by WIN 18,446 by further restricting Phenograph analysis to TCRβ^−^ populations. From the TCRβ^−^ concatenated sample, Phenograph identified 27 populations. After multiple *T* tests/FDR analysis, four populations were identified as discoveries (FDR adjusted *p*-values of *p* < 0.05) (Figure 4d and Figure S6). Three populations were increased in cells from the WIN 18,446-treated mice: CD103^+^ cells that did not express any other markers in the panel (Pheno 1), a CD11c^low^B220^+^MHCII^low^Ly6c^mid−hi^ population that is likely composed of plasmacytoid DCs (Pheno 24), and a Ly6c^low^ B cell population (Pheno 6). One population of DCs was decreased with treatment (Pheno 16).

## 4. Discussion

We previously reported that WIN 18,446 treatment increased survival and decreased colitis severity in *Smad3*^−/−^ mice in which *H. bilis* induced a rapid-onset (<7 days post-infection) severe colitis [23]. In this report, we show that WIN 18,446 treatment also inhibits colitis in a more chronic model of IBD—*Mdr1a*^−/−^ mice, but not in the *Il10^−/−^* mice. These results suggest that ALDH1A inhibition may be efficacious in certain types of IBD, similar to other existing treatments, and that IL-10 may be important for the WIN 18,446-mediated attenuation of colitis observed in *Mdr1a*^−/−^ and *Smad3*^−/−^ mice.

RA is a natural ligand for the nuclear transcription factors, retinoic acid receptors (RARs), that regulate the expression of a diverse array of genes, including those that encode the gut homing proteins, α4 and CCR9 [31]. Thus, cell types that produce RA can influence gut homing [3,8,9,32,33]. In cMLN of *Mdr1a*^−/−^ mice, we found increased cell numbers of various T cell subsets, while we observed decreased α4ß7 integrin expression on the same cell types. RA-producing DCs induce gut homing proteins on T cells primed in MLN [31]. WIN 18,446 treatment inhibits RA synthesis leading to reduced levels of α4ß7 integrin on T cells. Therefore, we suggest that T cell numbers may be increased in cMLN due to their reduced gut homing. Longitudinal studies will be needed to track cell numbers in cMLN and LPL of colon/cecum to gain insights into this process. Of note, the magnitude of the decrease in α4ß7 integrin expression was much larger in Th17 cells than other T cell types examined, indicating that the gut homing of Th17 cells could be more impacted by ALDH1A inhibition than the other T cell subsets. Because the Th17 pathway is significantly involved in colitis development in *Mdr1a^−/−^* mice [26], decreased homing of Th17 T cells to the gut may provide a plausible explanation for attenuated colitis in WIN 18,446-treated mice.

Though we suspect that altered Th17 cell trafficking and function play a role in reduced disease in WIN 18,446-treated *Mdr1a*^−/−^ mice, we did not observe changes in serum IL17F or IL17A associated with WIN 18,446 treatment or colitis severity. This may be due to the transient nature of IL17A increasing in serum in response to *H. bilis* infection in this model; IL17A was expressed at 2 weeks post-*H. bilis* infection, but not at 6.5 weeks post-infection [34]. Interestingly, IL17E (also known as IL25) and IL22 influence and are influenced by the Th17 and Th1 and regulatory axes (for reviews see [35,36]), and were both decreased in the serum of mice treated with WIN 18,446. As tuft cells in the intestinal epithelium produce the majority of IL25 [35], decreased IL25 in WIN 18,446-treated *Mdr1a*^−/−^ mice with less severe disease compared to mice with no treatment could reflect fewer tuft cells in a less hyperplastic and inflamed epithelial barrier [37]. IL22 is thought to have mainly a protective influence in IBD through its action on intestinal repair and recovery from inflammation [36]. Its decreased levels in serum could reflect decreased activities of Th17 in WIN 18,446-treated mice, as these cells are large producers of IL22 [36].

While we observed a reduction in α4ß7 expression on CD4^+^ T cells in both *Mdr1a^−/−^* mice *Smad3*^−/−^ mice [23], there were differences in immune cell changes between the two models in response to WIN 18446 treatment. An altered ratio of Th17 to iTreg cells was observed only in the *Smad3*^−/−^ mice [23], but not in *Mdr1a*^−/−^ mice. No changes in α4ß7 were observed in CD8^+^ T cells and the fold change in α4ß7 expression between WIN 18,446-treated and untreated mice was not different between the different CD4^+^ T cell subsets in the *Smad3*^−/−^ model, as was observed in *Mdr1a*^−/−^ mice. These differences are likely due to different genetic susceptibilities that cause colitis, and a different time course of colitis development (acute vs. chronic) in these two models.

In addition to decreased α4ß7 integrin expression with WIN 18,446 treatment in *Mdr1a*^−/−^ colitis, we observed increased percentages of cells expressing CD103 in all T cell subsets evaluated, with the magnitude of the change being highest in the Th17 cell subset. In the *Smad3*^−/−^ mouse model, we noticed that WIN 18,446-treated mice also have increased CD103^+^ T cells, though we have not evaluated CD103 expression on different T cell subsets in this model [38]. It has also been reported that CD4^+^ and CD8^+^ T cells expressing CD103 are elevated in heathy tissue compared to diseased tissue in human IBD [39,40]. Therefore, increased CD103^+^ T cells observed in WIN 18,446-treated mice may be important to the mechanism involved in reduced colitis severity that correlates with observations in human IBD. 

Phenograph analysis showed that WIN 18,446 treatment influences multiple immune cell subsets. For example, WIN 18,446 treatment differentially altered T cells expressing different levels of Ly6c. Ly6c, in combination with other markers, has been used to distinguish different subsets of memory and effector T cells in both CD8^+^ and CD4^+^ T cell responses [41,42,43], and may play a role in the migration of these T cell subsets to secondary lymphoid structures [42]. As Ly6c expression can be affected by the cytokine environment during T cell activation [41], it is possible that WIN 18,446-induced changes in inflammation affected Ly6c expression on certain T cell subsets, thus increasing their presence in cMLN. In addition, we observed changes in multiple non-T cell populations including in DC, pDCs and B cells. CD11b^+^ DCs with variable expression of CD103 were decreased in the cMLN of WIN 18,446-treated *Mdr1a*^−/−^ mice. We have made a similar observation in *Smad3*^−/−^ mice [38]. Others have noted the role of RA in the differentiation of CD11b^+^ DCs in the small intestinal lamina propria [44,45]. Vitamin A deficiency or use of a pan-RAR antagonist reduced intestinal CD103^+^CD11b^+^ DCs, while CD103^+^CD11b^−^ DCs were not affected or increased. This appeared to be driven by RA-associated signaling during transition from pre-DC to the CD11b^+^ DC subset [44]. As CD11b^+^ DCs (cDC2) are thought to be important in the induction of Th17 responses [46], a generalized reduction in this subset may lead to protection from colitis for conditions where Th17 responses are important to the disease process. Plasmacytoid DCs [47] (CD11c^low^B220^+^MHCII^low^Ly6c^mid−hi^) were increased with WIN 18,446 treatment. Others have shown that RA can induce the upregulation of α4ß7 integrin in peripheral blood pDC (and DC in general), and that this is also associated with the downregulation of CD62L that is involved in immune cells trafficking to lymph nodes [48]. It is possible that in the presence of reduced RA due to WIN 18,446 treatment, pDCs traffic to the MLN rather than to the lamina propria, as the upregulation of α4ß7 integrin and associated downregulation of CD62L would not occur. Based on these analyses, future studies will be designed to further refine subset identities and the importance of these changes to IBD. 

## 5. Conclusions

We have shown that the inhibition of ALDH1A enzymes by an orally deliverable small molecule is able to reduce the severity of clinical symptoms of colitis and histological inflammation in the colon/cecum in a mouse model of chronic IBD, *Mdr1a^−/−^* mice, when treatment is initiated prior to the onset of inflammation. The most consistent immune cell change associated with ALDH1A inhibition was decreased expression of α4ß7 integrin on activated T cells, particularly Th17 cells. This is of interest because antibodies against α4ß7 integrin are one of the more promising biologics in IBD treatment. We anticipate that ALDH1A inhibition has the potential to decrease the dose and/or frequency of α4ß7 antibody needed for IBD treatment, as it will decrease the expression of the protein that is targeted by the antibody. WIN 18,446 treatment also induced changes to immune cells including DCs, plasmacytoid DCs, and B cells that have not been previously studied in this context. If these are additional beneficial immunological changes that are different from those induced by anti-α4ß7 antibodies, then the use of both types of treatment could boost the efficacy of each, again leading to lower dosing or frequency of each treatment, thus reducing potential side effects. We plan to evaluate the efficacy of WIN 18,446 when administered during active colitis (rather than given before the colitis trigger) and to identify specific immune cell subsets that are altered in response to the inhibitor. Our studies are the first step in developing a new family of oral drugs to treat IBD. 

## Data Availability

Data are contained within the article and Supplementary Material. Flow cytometry data are available on request.

## Supplementary Materials

The following supporting information can be downloaded at: https://www.mdpi.com/article/10.3390/nu15183883/s1, Figure S1: Gating strategy for T cell subsets; Figure S2: ALDH1A inhibition by WIN 18,446 treatment did not reduce colitis severity in the *Il10*^−/−^ (BALB/c) mouse model of IBD; Figure S3: Percentages, cell numbers and a ratio of T cell subsets in cMLN of mice with and without WIN 18,446 treatment; Figure S4: Gating strategies used to prepare concatenated samples for the Phenograph and tSNE analyses; Figure S5: T cell populations altered with WIN 18,446 treatment identified using population-discovery methods; Figure S6: Histograms of marker expression levels of the Phenograph-defined populations shown in Figure 4.
