# Supplementary material for: ALDH1A Inhibition Suppresses Colitis and Alters α4β7 Integrin Expression on Activated T Cells in Mdr1a−/− Mice
Source: Nutrients. 2023 Sep 6;15(18):3883. doi: 10.3390/nu15183883 (PMC10536456; doi:10.3390/nu15183883)
Supplement: Supplementary file 1 [file nutrients-15-03883-s001.zip › nutrients-2541481-supplementary.pdf]

nutrients

MDPI

Article

ALDH1A Inhibition Suppresses Colitis and Alters α4β7 Integrin Expression on Activated T Cells in *Mdr1a*$^{-/-}$ Mice

Audrey Seamons, Olesya Staucean, Jessica M. Snyder, Thea Brabb, Charlie C. Hsu and Jisun Paik *

Supplementary Figures and Figure legends

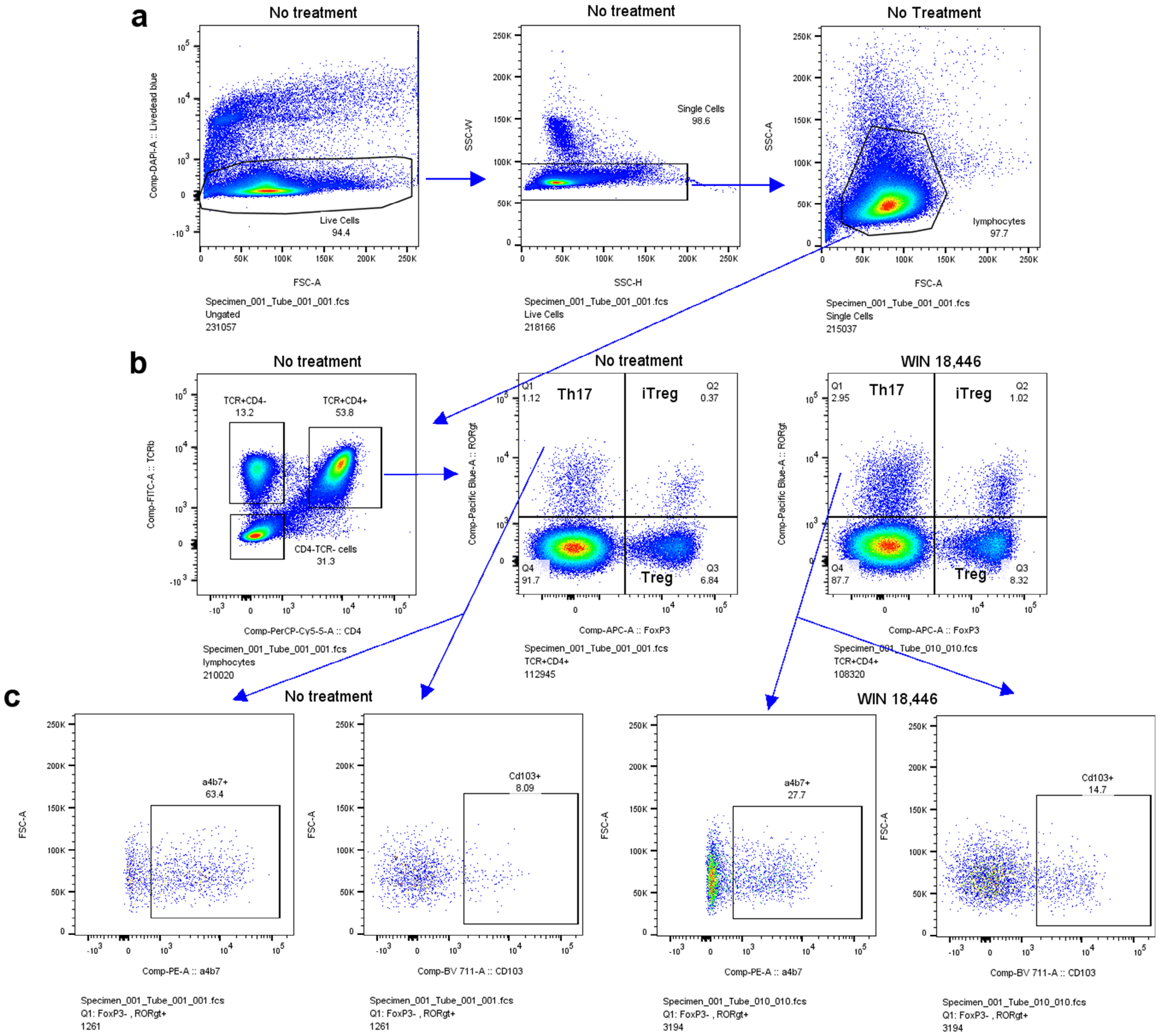

Figure S1. Gating strategy for T cell subsets. Populations were gated to remove dead cells (FSC-A vs. Live/Dead Blue—DAPI channel), doublets (SSC-H v. SSC-W), and then size exclusion (FSC-A vs SSC-A) was used to select lymphocytes (**a**). CD4$^+$ and CD4$^-$ T cells were defined (B, left panels) and were further characterized using FoxP3 vs. RORγt expression (**b**, center and right panels). Example gates for α4β7 integrin and CD103 are shown for Th17 T cells (**c**). The treatment group of the example is indicated in the figure above the panels.

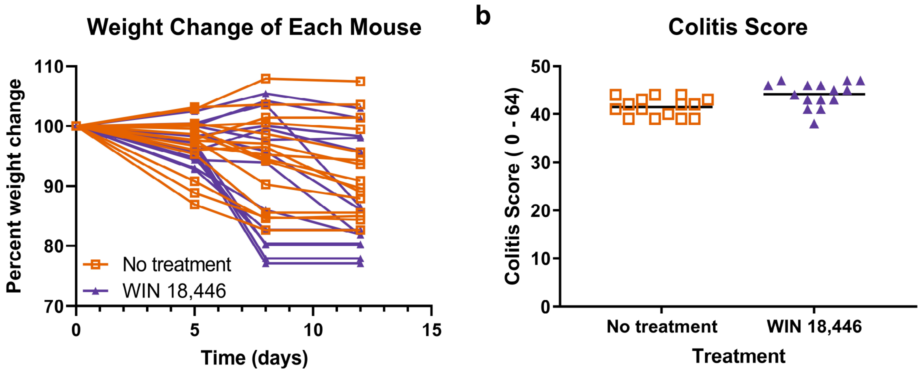

Figure S2. ALDH1A inhibition by WIN 18,446 treatment did not reduce colitis severity in the *Il10*$^{-/-}$ (BALB/c) mouse model of IBD. Male mice were treated with and without WIN 18,446 (2 mg/g diet) one week prior to *H. bilis* inoculation; treatment was continued over the entire experiment. Mice were weighed weekly (**a**) and were euthanized based on predetermined criteria. Colitis severity scores were determined by histological analysis (**b**). Horizontal bar is the mean colitis score.

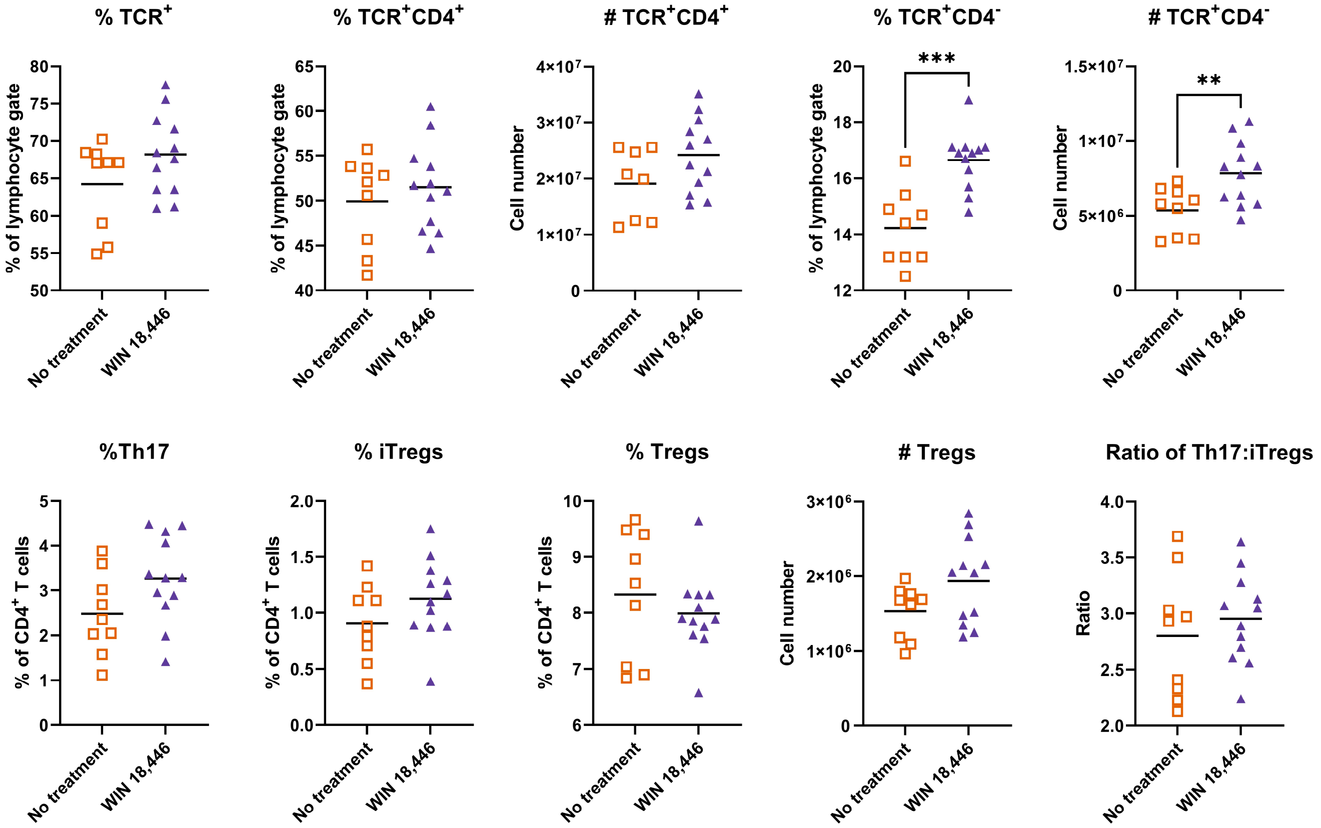

Figure S3. Percentages, cell numbers and a ratio of T cell subsets in cMLN of mice with and without WIN 18,446 treatment. Presented values of T cell subsets are associated with data shown in Figure 3. Statistical significance (unadjusted, unpaired t test with Welch's correction) is indicated. **P<0.01, ***P<0.001, ****P<0.0001.

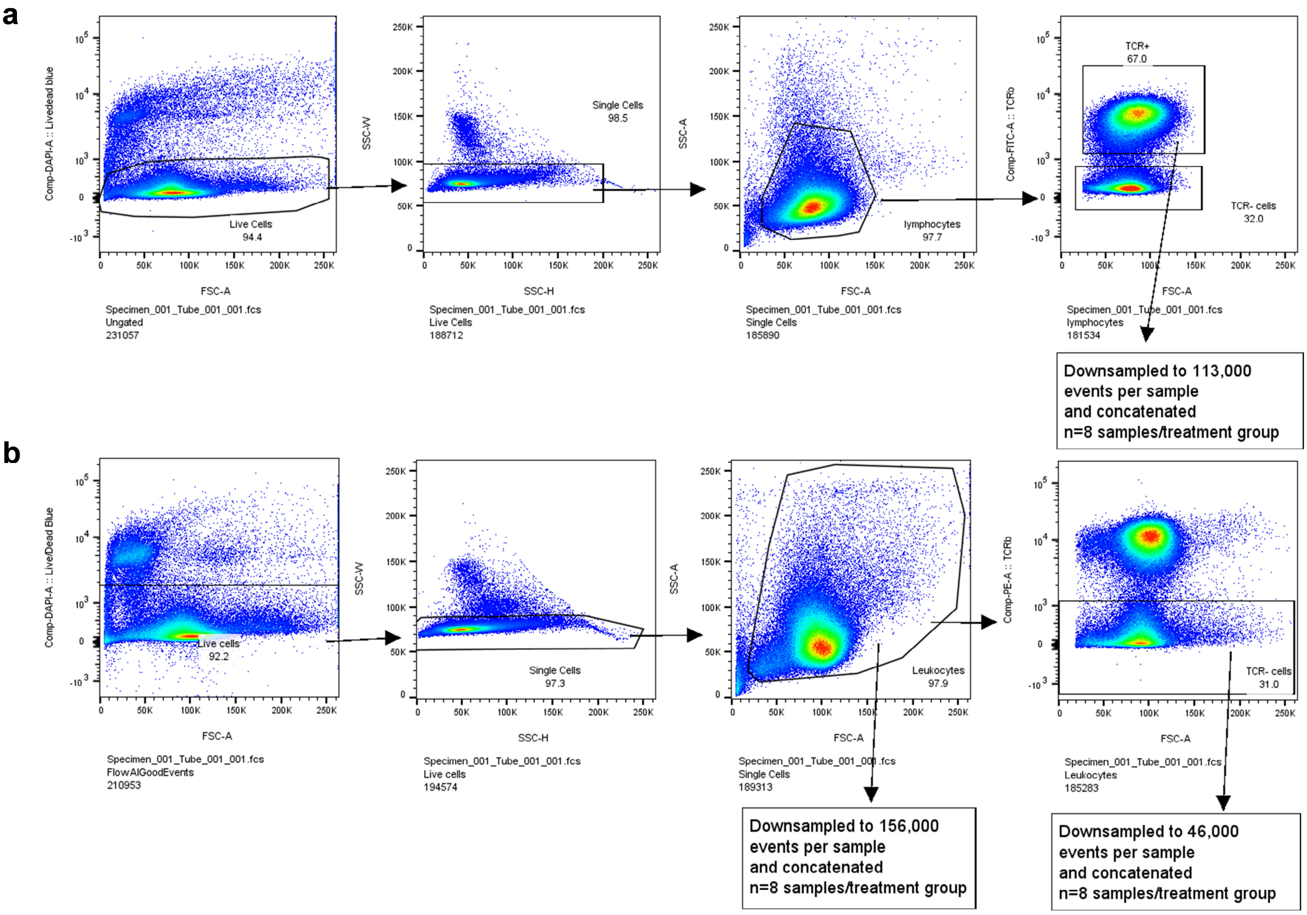

Figure S4. Gating strategies used to prepare concatenated samples for the Phenograph and tSNE analyses. Dead cells and doublets were excluded based on Live/Dead Blue staining and SSC-W vs. SSC-H parameters, respectively (**a** and **b**). A lymphocyte (**a**) or larger leukocyte (**b**) gate was created based on FSC-A and SSC-A parameters. TCRβ$^+$ cells were subsampled using the DOWNSAMPLE plugin to 113,000 per sample from the lymphocyte gate (**a**). Leukocytes (156,000 cells per sample) and TCRβ$^-$ leukocytes (46,000 per sample) were also subsampled (**b**). Equivalent samples per treatment group (n=8 samples/treatment, two treatment groups for 16 total samples) and cell numbers per subsample were concatenated for each analysis.

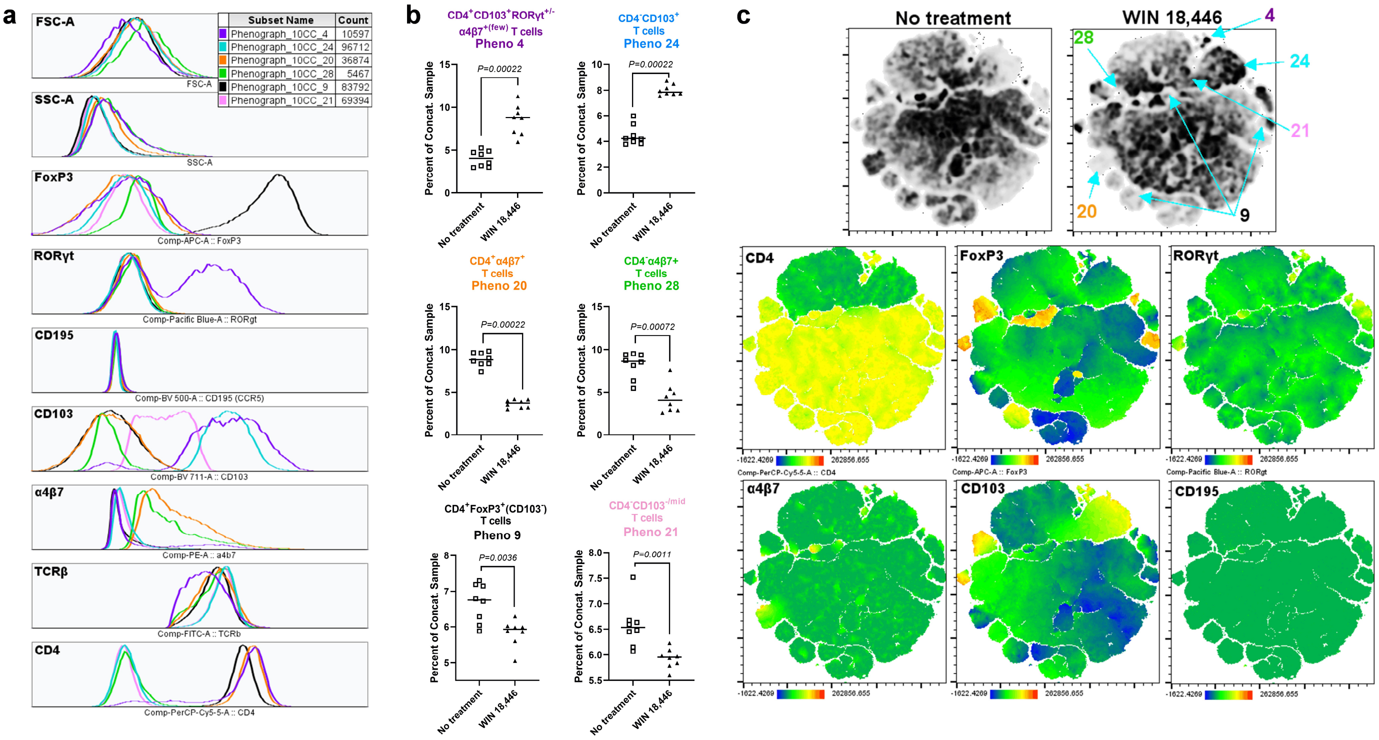

Figure S5. T cell populations altered with WIN 18,446 treatment identified using population-discovery methods. T cells from equal numbers of samples per treatment group were concatenated (n=8 samples/treatment, total 16 samples) and Phenograph analysis performed. The percent of the concatenated sample comprised of cells from each individual sample was determined and compared by treatment group. Six of 31 Phenograph-defined populations were significantly different between treatment groups based on markers other than TCRβ and CD4: Pheno 4, Pheno 24, Pheno 20, Pheno 28, Pheno 9 and Pheno 21. Expression profiles of the six populations are shown using color-coded histograms (**a**). Percent contribution of individual samples to the concatenated sample is plotted by treatment group for the same six populations with FDR-adjusted *p*-values (5%) indicated (**b**). Dimensionality reduction analysis (tSNE) generated plots representing immune cell subsets in two dimensions (**c**). Density plots of the concatenated sample were produced for no treatment and WIN 18,446 groups (**c**, top row) and show global increases (darker areas) or decreases (lighter areas) in cell densities. Blue arrows indicate changes in density correlating to phenotypes of the Phenograph-defined populations in (**a**) and (**b**). Heat maps (**c**, middle and bottom rows) show locations of immune cell populations expressing each fluorescent marker on the two-dimensional plots.

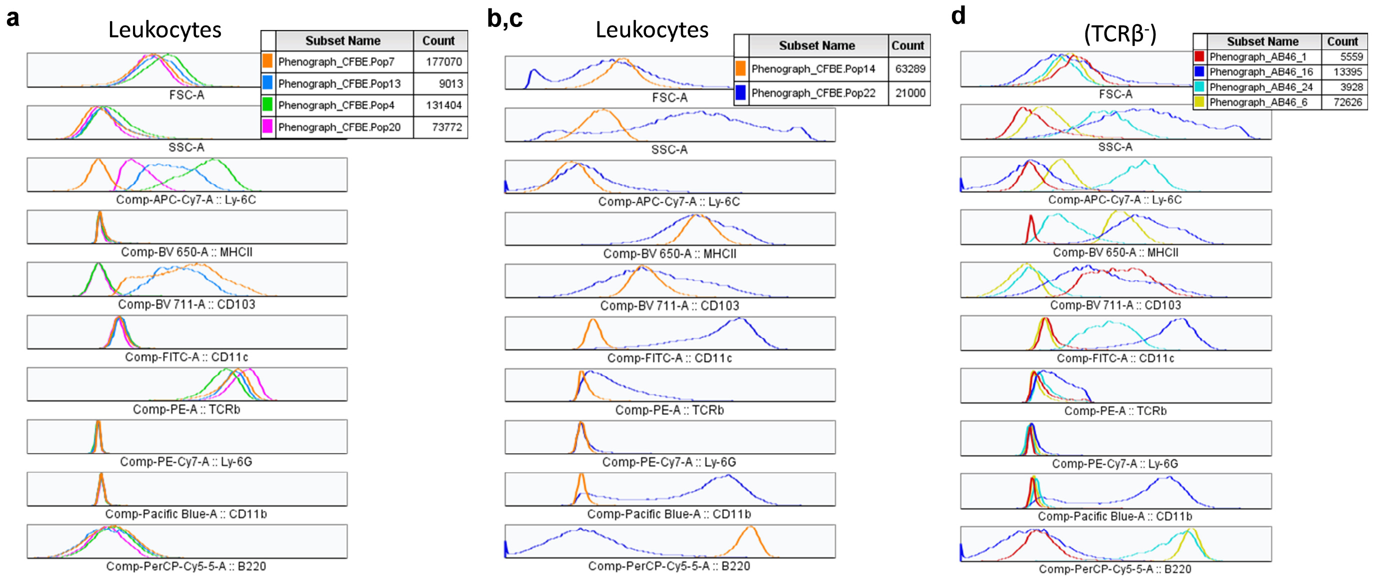

Figure S6. Histograms of marker expression levels of the Phenograph-defined populations shown in Figure 4. From the concatenated sample of leukocytes, four T cell populations with varying expression levels of Ly-6c and CD103 (**a**), and a B cell and dendritic cell population (**b**, **c**) were identified. The four populations identified from the concatenated TCRβ⁻ leukocytes Phenograph analysis are shown in (**d**).
